# Effectiveness of Pilates exercise in treating people with chronic low back pain: a systematic review of systematic reviews

**DOI:** 10.1186/1471-2288-13-7

**Published:** 2013-01-19

**Authors:** Cherie Wells, Gregory S Kolt, Paul Marshall, Bridget Hill, Andrea Bialocerkowski

**Affiliations:** 1 School of Science and Health, University of Western Sydney, Locked Bag 1797, Penrith, NSW, 2751, Australia; 2 Griffith Health Institute, Griffith University, Gold Coast, Qld, 4222, Australia

**Keywords:** Pilates, Exercise, Low back pain, Systematic review

## Abstract

**Background:**

Systematic reviews provide clinical practice recommendations that are based on evaluation of primary evidence. When systematic reviews with the same aims have different conclusions, it is difficult to ascertain which review reported the most credible and robust findings.

**Methods:**

This study examined five systematic reviews that have investigated the effectiveness of Pilates exercise in people with chronic low back pain. A four-stage process was used to interpret findings of the reviews. This process included comparison of research questions, included primary studies, and the level and quality of evidence of systematic reviews. Two independent reviewers assessed the level of evidence and the methodological quality of systematic reviews, using the National Health and Medical Research Council hierarchy of evidence, and the Revised Assessment of Multiple Systematic Reviews respectively. Any disagreements were resolved by a third researcher.

**Results:**

A high level of consensus was achieved between the reviewers. Conflicting findings were reported by the five systematic reviews regarding the effectiveness of Pilates in reducing pain and disability in people with chronic low back pain. Authors of the systematic reviews included primary studies that did not match their questions in relation to treatment or population characteristics. A total of ten primary studies were identified across five systematic reviews. Only two of the primary studies were included in all of the reviews due to different inclusion criteria relating to publication date and status, definition of Pilates, and methodological quality. The level of evidence of reviews was low due to the methodological design of the primary studies. The methodological quality of reviews varied. Those which conducted a meta-analysis obtained higher scores.

**Conclusion:**

There is inconclusive evidence that Pilates is effective in reducing pain and disability in people with chronic low back pain. This is due to the small number and poor methodological quality of primary studies. The Revised Assessment of Multiple Systematic Reviews provides a useful method of appraising the methodological quality of systematic reviews. Individual item scores, however, should be examined in addition to total scores, so that significant methodological flaws of systematic reviews are not missed, and results are interpreted appropriately. (348 words)

## Background

Systematic reviews are ranked as the most valid form of research in several hierarchies of evidence [1,2]. They provide evidence-based recommendations from the synthesis and critically appraisal of primary studies [3]. Within health care, systematic reviews are used to efficiently obtain advice regarding client management [4]. Conflicting results of systematic reviews, however, creates confusion for readers [5].

Several recently published systematic reviews have investigated the effectiveness of Pilates in people with chronic low back pain (CLBP) [6-10]. Pilates is a mind-body exercise that targets core stability, strength, flexibility, posture, breathing, and muscle control [11]. It has been recommended in the management of people with CLBP, as this type of exercise may strengthen deep, stabilising muscles that support the lumbar spine, such as transverses abdominis [6,12]. These muscles are inhibited in people with CLBP [13,14].

Reviews examining the efficacy of Pilates in people with CLBP, however, report different conclusions. La Touche et al. (2008) [6] suggested that Pilates reduces pain and disability, while Lim et al. (2011) [7] reported that Pilates reduces pain when compared to minimal treatments, but not disability. In contrast, Pereira et al. (2012) [8] concluded that Pilates is ineffective in reducing pain and disability, and Posadzki et al. (2011) [9] suggested that evidence was inconclusive. Aladro-Gonzalvo et al. (2012) also provided conflicting results reporting that Pilates may reduce pain only when compared to minimal intervention, and disability only when compared to other physiotherapeutic treatments [10]. These contradictory findings make it difficult to conclude on the efficacy of Pilates in people with CLBP and to direct use in clinical settings.

A systematic review of reviews was conducted to critically evaluate and summarise the results of all published systematic reviews that have investigated the effectiveness of Pilates exercise in reducing pain and disability in people with CLBP. Areas for improvement for systematic reviews were subsequently identified, and an evidence-based conclusion provided regarding the efficacy of Pilates exercise in people with CLBP.

## Methods

**Figure 1:**
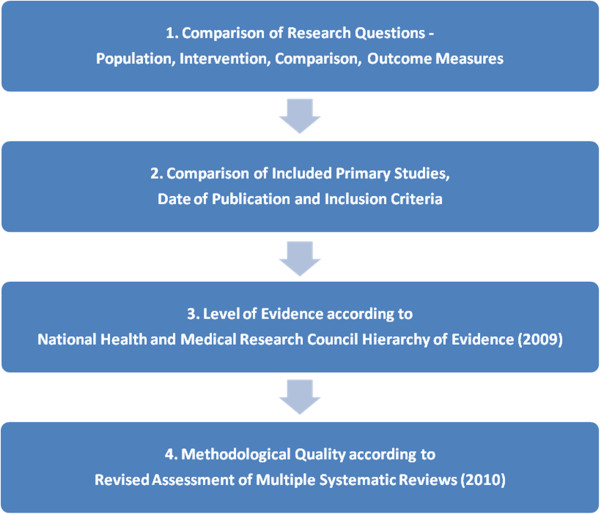
Review process for systematic reviews.

A four-stage process was used to determine the appropriateness of systematic review conclusions. This involved comparison of reviews with respect to research questions, included primary studies, their level of evidence and methodological quality (Figure 1). The level of quality of the reviews was assessed using the National Health and Medical Research Council hierarchy of evidence (2009) [1], while the methodological quality was assessed using the Revised Assessment of Multiple Systematic Reviews (R-AMSTAR) [15]. Systematic review findings were then interpreted with respect to these factors.

### Study design

A systematic review design was chosen over a narrative review as it limits bias in the selection and appraisal of evidence [16-18]. In a systematic review, a comprehensive search of the literature is undertaken to answer a focused research question; the search strategy, criterion for selection and critical appraisal of literature is defined; quantitative rather than qualitative results are reported and evidence-based inferences are made [18]. This systematic review was written to meet Preferred Reporting Items for Systematic Review and Meta-Analyses (PRISMA) guidelines [3].

### Search strategy

A comprehensive literature search was undertaken using ten databases including Cumulative Index to Nursing and Allied Health Literature (CINAHL), Cochrane Library, Medline, Physiotherapy Evidence Database (PEDro), ProQuest:Health and Medical Complete, Proquest: Nursing and Allied Health Source, Proquest Research Library: Health and Medicine, Scopus, Sport Discus, and Web of Science. The standardised search strategy included the use of Medical Subject Headings (MeSH) terms “Pilates” and “Low Back Pain”, and search term “Review” in the title, abstract, and as able, the keyword fields within maximal date ranges of each database up until November 4, 2012 (Table 1).

**Table 1:** Search strategy: using medical search headings (MeSH) “Pilates” and “Low Back Pain”, and search term “Review”

**Database**	**Date Range**	**Fields**
Cumulative Index to Nursing and Allied Health Literature (CINAHL)	1970–2012	Title, Abstract, or Word in Subject Heading
Cochrane Library	1800–2012	Title, Abstract or Keyword
Medline	1928–2012	Title, Abstract or Keyword
Physiotherapy Evidence Database (PEDro)	1928–2012	Title and Abstract
Proquest		
Medical and Health Complete	1928–2012	Title, Abstract, or Subject Heading
Nursing and Allied Health Source		
Research Library		
Scopus	1960–2012	Title, Abstract, or Keyword
Sport Discus	1975–2012	Title, Abstract, or Keyword
Web of Science	1977–2012	Topic or Title

Preliminary searching revealed that expanding the search to include “exercise”, “motor control”, and “core stability” did not identify any additional reviews, nor did changing the Boolean operator to “or”. Removing “Low Back Pain” and “Review” also did not help identify any additional systematic reviews. Secondary searching of reference lists of included papers was undertaken to identify any additional, relevant studies that met the inclusion criteria.

### Selection procedures

Selection of relevant papers was based on the title, and if required, review of the abstract or full text of the document. Papers identified from the search process were assessed against inclusion and exclusion criteria by two independent reviewers (CW, BH). If there were any discrepancies in selected papers between the two reviewers, a third reviewer (AB) independently reviewed the papers and through discussion, obtained a consensus.

### Selection criteria

To be included in this systematic review, systematic reviews needed to:

· Be identified as a systematic review of 2 or more intervention studies. In a systematic review, a comprehensive search of the literature is undertaken to answer a focused research question; the search strategy, criterion for selection and critical appraisal of literature is defined; quantitative rather than qualitative results are reported and evidence-based inferences are made [16,17]. Narrative reviews or expert commentaries did not meet inclusion requirements [17].

· Be published in the English language. For ease of interpretation and access, reviews that were unpublished or published in another language were excluded.

· Include human participants with chronic low back pain, that is, localised pain in the lumbar region that lasts for more than three months [19]. If reviews only included participants with low back pain lasting less than three months, they were excluded.

· Assess the effectiveness of Pilates, where the term “Pilates” was used to describe the type of prescribed exercise being investigated. Exercises described as “motor control” or “lumbar stabilisation” did not suffice for Pilates. This is because Pilates may include features in addition to these exercise approaches [11].

· Use outcome measures to evaluate disability, that is, impairments, activity limitations or participation restrictions according to the International Classification of Health, Functioning, and Disability (ICF) [20]. Pain is considered a functional impairment in the ICF.

### Level of evidence

According to the NHMRC hierarchy, the level of evidence of a systematic review depends on the methodological design of included primary studies [1]. Systematic reviews that include only randomised controlled trials are rated as the highest form of evidence. Systematic reviews that include studies other than randomised controlled trials are rated only as high as the lowest level of evidence represented by primary studies (Table 2). Two independent reviewers graded the level of evidence of systematic reviews according to the NHRMC hierarchy of evidence [1]. Any discrepancies between the two reviewers were discussed with a third reviewer to obtain a consensus.

**Table 2:** Modified national health and medical research council (NHMRC) hierarchy of evidence

**Level**	**Type of Intervention**
I	Systematic Review of Randomised Controlled Trials
II	Randomised Controlled Trial
III	Pseudo-Randomised Controlled Trial, Comparative Study with or without Concurrent Controls
IV	Case Series with either post-test or pre-test/post-test outcomes

* Adapted from NHMRC (2009) [1].

### Methodological quality

The methodological quality of included systematic reviews was evaluated using the R-AMSTAR [15]. The R-AMSTAR rates the methodological quality of systematic reviews by providing a numerical score for 11 items (Table 3). For each item, the methodological quality is scored out of 4 where one indicates poor methodological quality, and four indicates excellent methodological quality [15]. R-AMSTAR items originate from the Assessment of Multiple Systematic Reviews (AMSTAR). While the AMSTAR has been shown to be valid and reliable in assessing the methodological quality of reviews, the numerical score provided by the R-AMSTAR provides an additional quantitative score that is easy to interpret [15,21,22].

**Table 3:** R-AMSTAR scores for methodological quality of systematic reviews

**Systematic Review**	R-AMSTAR Scores Per Criterion (/4)*											**R-AMSTAR Total (/44)**
	**1**	**2**	**3**	**4**	**5**	**6**	**7**	**8**	**9**	**10**	**11**	
Aladro-Gonzalvo et al. 2012 [10]	4	4	3	3	4	4	4	4	3	3	2	**37**
La Touche et al. (2008) [6]	3	2	2	1	1	3	3	1	1	1	1	**19**
Lim et al. (2011) [7]	4	2	4	3	4	4	3	4	3	3	1	**35**
Pereira et al. (2012) [8]	3	4	4	3	2	2	4	2	4	2	2	**32**
Posadzki et al. (2011) [9]	3	4	3	4	4	2	3	4	1	1	1	**30**
* Note:												
**R-AMSTAR Item**	**Description**											
1.	Was an ‘a priori’ design provided?											
2.	Was there duplicate study selection and data extraction?											
3.	Was a comprehensive literature search performed?											
4.	Was the status of publication (i.e. grey literature) used as an inclusion criterion?											
5.	Was a list of studies (included and excluded) provided?											
6.	Were the characteristics of the included studies provided?											
7.	Was the scientific quality of the included studies assessed and documented?											
8.	Was the scientific quality of the included studies used appropriately in formulating conclusions?											
9.	Were the methods used to combine the findings of studies appropriate?											
10.	Was the likelihood of publication bias (a.k.a. “file drawer” effect) assessed?											
11.	Was the conflict of interest stated?											
**R-AMSTAR Score**	**Interpretation**											
1	Score if satisfied 0 of the criteria [Items 1,2,4,6,10,11] or 0 or 1 of the criteria [Items 3,5, 7–9]											
2	Score if satisfied 1 of the criteria [Items 1,2,4,6,10,11] or 2 of the criteria [Items 3,5, 7–9]											
3	Score if satisfied 2 of the criteria [Items 1,2,4,6,10,11] or 3 of the criteria [Items 3,5, 7–9]											
4	Score if satisfies 3 of the criteria [Items 1,2,4,6,10,11] or 4 of the criteria [Items 3,5, 7–9]											

Adapted from Kung, Chiappelli, Cajulis, Avezova, Kossan, 2010 [15].

Two independent reviewers graded the reviews, with any discrepancies being resolved by discussion with a third reviewer. R-AMSTAR items were graded as per guidelines provided by Kung et al. (2010) [15]. Percentile ranks were not calculated in this systematic review due to the small number of reviews being considered. Following grading of the methodological quality of the three systematic reviews, the percentage agreement and kappa score of agreement, and 95% confidence interval, between the two independent reviewers were calculated.

### Data extraction and syntheses

The following data were extracted and synthesised from selected papers:

1. Author(s), year of publication, and reference of systematic reviews. Descriptive statistics were used to summarise the number of systematic reviews and dates of publication.

2. The findings and conclusions of systematic reviews in relation to pain and disability, including effect sizes and 95% confidence intervals provided by meta-analyses.

3. Author(s), year of publication, and reference of primary studies included in the systematic reviews. Descriptive statistics were used to summarise the number of primary studies, and differences in included primary studies across systematic reviews.

4. The NHMRC level of evidence and R-AMSTAR scores for methodological quality were calculated for each review and tabulated alongside author(s) and year of publication.

5. The research questions of systematic reviews in terms of study population, intervention, comparisons, and outcome measures. This included consideration of systematic review aims, and corresponding included primary study details.

## Results

**Figure 2:**
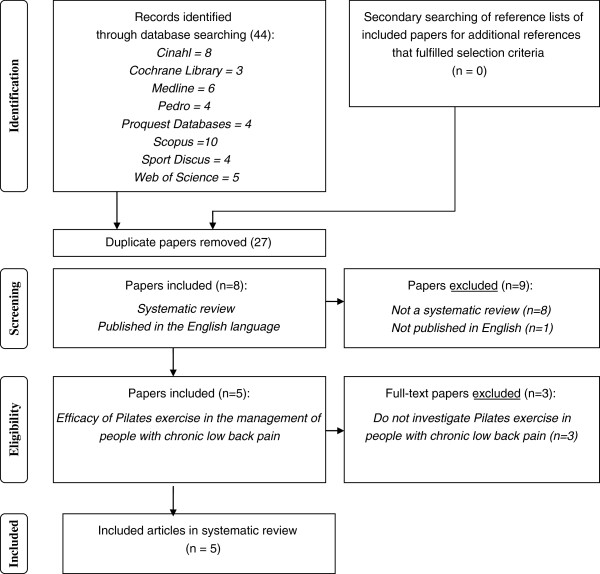
Results of literature search.

A total of 44 papers were identified using the search strategy described in the methods. Five of these papers fulfilled selection criteria [6-10]. There was 100% agreement among the two independent reviewers on the selection of the systematic reviews. Most papers were excluded due to being duplicates, or not using a systematic review methodology (Figure 2).

### Findings of systematic reviews

The five reviews had conflicting conclusions regarding the effectiveness of Pilates in reducing pain and disability in people with CLBP (Table 7). Three of the reviews conducted meta-analyses [7,8,10]. Aladro-Gonzalvo et al. (2012) [10] also conducted a meta-regression analysis to identify co-variants that may have contributed to the heterogeneity of treatment effect across studies [23]. No predictor variable, however, was identified.

### Research questions

a) Population

The authors of all reviews, apart from Posadzki et al. (2011) [9], failed to ensure the duration of symptoms reported by participants in primary studies matched with their research questions. For example, La Touche et al. (2011) [6] and Pereira et al. (2012) [8] aimed to focus on participants with CLBP, and Aladro-Gonzalvo et al. (2012) [10] and Lim et al. (2011) [7] on participants with low back pain lasting more than 6 weeks. The authors of these reviews, however, included primary studies with participants with acute, subacute, recurrent or chronic low back pain (Table 4).

b) Intervention

Diverse Pilates exercise protocols for people with low back pain were reported across reviews (Table 4). In the majority of primary studies, authors prescribed Pilates mat exercises, although Anderson (2005) [24] and Rydeard et al. (2006) [25] suggested use of specialised Pilates equipment. Only 60% of primary studies described home exercises as part of the Pilates protocol [24-29].

The validity of Pilates exercise interventions in reviews also varied. La Touche et al. (2008) [6], Lim et al. (2011) [7], Pereira et al. (2012) [8], and Aladro-Gonzalvo et al. (2012) [10], ensured that treatments in primary studies were described solely as Pilates exercise. Posadzki et al. (2011) [9], however, included a primary study where treatment involved yoga, rehabilitation, and physical therapy as well [30].

c) Comparison

Comparison treatments varied considerably, ranging from no exercise, usual care, massage, physiotherapy, and alternative exercises (Table 4). Usual care comparison treatments also differed, ranging from education and medication, to physiotherapy and bracing [25,30,31]. Co-interventions were also evident in two primary studies [29,31].

There was also inconsistency across reviews regarding the description of comparison physiotherapy treatment within the Obrien et al. 2006 [32] study. Pereira et al. (2012) [8] defined the type of physiotherapy as lumbar stabilisation exercise, while Lim et al. (2006) [7] reported that the physiotherapy treatment included other modalities as well.

d) Outcome measures

Similar outcome measures were used across primary studies and in the systematic reviews (Table 4). Lim et al. (2011) [7], Aladro-Gonzalvo et al. (2012) [10], and Pereira et al. (2012) [8], however, elected to use different outcome measures for pain given in the same primary study (Anderson, 2005) [24]. That is, Lim et al. (2011) [7] and Aladro-Gonzalvo et al. (2012) [10] used the Miami Back Pain Index scores [33], while Pereira et al. (2012) used pain scores given within the Short Form Health Survey (SF-36) [34].

Although similar outcome measures were used across reviews, participants were evaluated at different points in time across primary studies. Timing of evaluation was dependent on the duration of the Pilates treatment and the length of follow up. The shortest follow up was at 6 weeks [28,31,32] and longest follow up assessment was at 12 months following the completion of Pilates treatment [24,25,30].

**Table 4:** Description of population, intervention, comparison, outcomes measures in systematic reviews

**Systematic Review**	**Population**	**Intervention**	**Comparison**	**Outcome Measures**
1. Aladro-Gonzalvo et al. (2012) [10]	Nonspecific low back pain greater than 6 weeks or recurrent (twice per year)	60 minute sessions	Usual care, normal exercise or sports, back school exercise^+^, lumbar stabilisation exercise, massage, physiotherapy	*Pain:* MBI–pain, NRS-101, VAS
		1–7 sessions/week		*Disability:* ODQ, RMDQ
		10 days −12 weeks		
2. La Touche et al. (2008) [6]	Nonspecific low back pain greater than 6 weeks or recurrent (twice/year)	50–60 minute sessions	Usual care, back school exercise^+^	*Pain:* NRS–101, RMVAS, VAS
		1–7 sessions/week		*Disability:* ODQ, RMDQ
		10 days–6 weeks		
3. Lim et al. (2011) [7]	Non specific low back pain of any duration or recurrence rate	30–60 minute sessions	Usual care, no exercise, back school exercise ^+^, lumbar stabilisation exercise, massage, physiotherapy	*Pain:* MBI–pain, NRS-101, RMVAS, VAS
		1–7 sessions/week		
				*Disability:* ODI, ODQ, RMDQ
		10 days–12 weeks		
4. Pereira et al. (2012) [8]	Non specific low back pain of any duration or recurrence rate	30–60 minute sessions	Usual care, lumbar stabilisation exercise massage, physiotherapy	*Pain:* SF-36 Pain, NRS–101, RMVAS, VAS
		1–3 sessions/week		
				*Disability:* ODI, ODQ, RMDQ
		4–8 weeks		
5. Posadzki et al. (2011) [9]	Nonspecific low back pain greater than 6 weeks or recurrent (twice/year); specific low back pain with disc pathology greater than 6 weeks	15–60 minute sessions	Usual care, back school exercise^+^	*Pain:* NRS−11, NRS–101, RMVAS, VAS
		1–7 sessions/week		*Disability:* ODQ, RMDQ
		10 days–12 months		

Abbreviations: MBI-pain - Miami Back Index pain subscale; NRS −11 - 11 point Numeric Rating Scale; NRS −101 - 101 point Numeric Rating Scale; ODI - Oswestry Disability Index; ODQ - Oswestry Low Back Pain Questionnaire; RMDQ - Roland Morris Disability Questionnaire; RMVAS -Roland Morris Visual Analog Scale; SF-36 Pain - Short Form Health Survey – Pain; VAS - Visual Analog Scale.

*+* Back school exercise includes respiratory and postural education, muscle strengthening and mobilisation exercise [7,23].

### Included primary studies

There were ten different primary studies identified across the five systematic reviews [24-32,35] (Table 5). La Touche et al. (2008) [6] and Posadzki et al. (2011) [9] included only studies published in full, as opposed to Aladro-Gonzalvo et al. (2012) [10], Lim et al. (2011) [7], and Pereira et al. (2012) [8] who included studies that were unpublished, or part-published [24,28,29,32,35]. Pereira et al. (2012) [8] also only included studies that had low risk of bias as defined by the Cochrane Back Review Group [36]. This meant that Donzelli et al. (2006) [27] and Quinn (2005) [35] were not included in this review.

**Table 5:** Primary studies included in systematic reviews

			**Primary Studies**							
**Systematic Reviews**	Donzelli et al. 2006 [27]	Gladwell et al. 2006 [31]	da Fonseca et al. 2009 [26]	Rydeard et al. 2006 [25]	Vad et al. 2007 [30]^+^	Anderson 2005 [24]*	Gagnon 2005 [28]*	MacIntyre 2006 [29]*	Quinn 2005 [35]*	O’Brien et al. 2006 [32] **
1. Aladro-Gonzalvo et al. 2012 [10]	√	√	√	√		√	√	√	√	√
2. La Touche et al. 2008 [6]	√	√		√						
3. Lim et al. 2011 [7]	√	√		√		√	√		√	√
4. Pereira et al. 2012 [8]		√		√		√	√			√
5. Posadzki et al. 2011 [9]	√	√		√	√					

+ Part-Pilates intervention.

* Unpublished theses.

** Abstract only.

### Level of evidence

There was 100% agreement between reviewers regarding the methodological design, and level of evidence of the primary studies and the systematic reviews. Primary studies consisted of randomised controlled trials (n=4), pseudo-randomised controlled trials (n=5), and a parallel case series (n=1). According to the National Health and Medical Research Council (NHMRC) hierarchy, the level of evidence represented by these primary studies ranges from Level II to Level IV evidence [1] (Table 6).

**Table 6:** Primary studies: level of evidence and methodological design

**NHMRC Level of Evidence**	**Methodological Design**	**Primary Studies**
II	Randomised Controlled Trial (n=4)	Anderson (2005) [24]*
		Gagnon (2005) [28]*
		MacIntyre (2006) [29]*
		Rydeard et al. (2006) [25]
III	Pseudo-Randomised Controlled Trial (n=5)	da Fonseca et al. (2009) [26]
		Gladwell et al. (2006) [31]
		O’Brien et al. (2006) [32]**
		Quinn (2005) [35]*
		Vad et al. (2007) [30]
IV	Parallel Case Series (n=1)	Donzelli et al. (2006) [27]

* Unpublished theses.

** Abstract only.

Aladro-Gonzalvo et al. (2012) [10], La Touche et al. (2008) [6], Lim et al. (2011) [7], and Posadzki et al. (2011) [9] included Donzelli et al. (2006) [27], a parallel case series article. These three reviews consequently represent Level IV evidence on the NHMRC hierarchy [1]. Pereira et al. (2012) [8] excluded Donzelli et al. (2006) [27], but included two pseudo-randomised controlled trials [31,32]. This means that the systematic review by Pereira et al. (2012) [8] represents Level III evidence on the NHMRC hierarchy [1].

### Methodological quality

The two reviewers agreed on 84% of R-AMSTAR scores across the systematic reviews (46/55). Different scores were obtained for criterion 9 and 10 for Aladro-Gonzalvo et al. (2012) [10], criterion 1, 2 and 6 for La Touche et al. (2008) [6], criterion 3 for Lim et al. (2011) [7], criterion 7 and 9 for Pereira et al. (2012) [8], criterion 8 for Posadzki et al. (2011) [9]. The inter-rater agreement for R-AMSTAR scores remained “substantial” when chance agreement was eliminated (kappa: 0.78, 95% confidence interval: 0.71-0.85) [37]. All disagreements were resolved through discussion with a third reviewer.

The R-AMSTAR scores of methodological quality of systematic reviews ranged from 19–37 out of 44 (Table 3). Aladro-Gonzalvo et al. (2012) [10] achieved the highest total score (37/44), followed by Lim et al. (2011) [7] (35/44), Pereira et al. (2012) [8] (32/44), Posadzki et al. (2011) [9] (30/44), and La Touche et al. (2008) [6] (19/44). The R-AMSTAR scores indicate that all reviews lacked a thorough assessment of publication bias and statement regarding conflict of interest. Duplicate data selection and extraction, inclusion of grey literature, listing of excluded studies, and documentation of study characteristics were also insufficient in several reviews [6-10].

Finally, R-AMSTAR scores identified that La Touche et al. (2008) [6] and Pereira et al. (2012) [8] needed to improve consideration of the methodological quality of the primary studies when formulating conclusions. Also, La Touche et al. (2008) [6] and Posadzki et al. (2011) [9] did not provide a justification for not undertaking a meta-analysis, and Lim et al. 2011 [7] and Aladro-Gonzalvo et al. (2012) [10] needed to improve their method of combining findings of primary studies in their meta-analyses.

## Discussion

This systematic review identified five published reviews that have investigated the efficacy of Pilates exercise in the treatment of people with CLBP [6-10]. These reviews have different conclusions, despite having similar research aims. To interpret results of reviews, a comparison of research questions, included primary studies, the level of evidence, and the methodological quality of systematic reviews was undertaken (Figure 1). This process assisted in identifying and understanding the reasons for the different review findings, and in considering the validity of those findings [36].

### Research questions

La Touche et al. (2008) [6] and Posadzki et al. (2011) [9] included primary studies with participants with sub acute, chronic or recurrent low back pain. Meanwhile, Aladro-Gonzalvo et al. (2012) [10], Lim et al. (2011) [7] and Pereira et al. (2012) [8] incorporated an additional primary study that included participants with acute low back pain as well [28]. Outcomes reported by and Aladro-Gonzalvo et al. (2012) [10], Lim et al. (2011) [7] and Pereira et al. (2012)[8] therefore may be conservative and underestimate the effects of Pilates in people with CLBP, as people with acute low back pain tend to respond less favourably to exercise [38].

The findings of Aladro-Gonzalvo et al. (2012) [10], La Touche et al. (2008) [6], Lim et al. (2011) [7], and Pereira et al. (2012) [8] relate to people with non-specific low back pain. Non-specific low back pain is pain in the lower back without an identifiable pathology [39]. In contrast, Posadski et al. (2011) [9] included an additional primary study with participants with low back pain related to disc pathology in the lumbar spine [30]. Further research into the effectiveness of Pilates in people with low back pain with specific pathologies should be undertaken so that conclusions can be made regarding the efficacy Pilates in people with all forms of low back pain [36].

With regards to treatment, Aladro-Gonzalvo et al. (2012) [10], La Touche et al. (2008) [6], Lim et al. (2011) [7], and Pereira et al. (2012) [8] included primary studies that investigated only Pilates exercise. Posadzki et al. (2011) [9], however, included a primary study that evaluated the effectiveness of an intervention that was only part-Pilates [30]. Treatment effects reported by this review may consequently relate to other therapies provided other than Pilates to the intervention group [40].

Pilates exercise protocols varied considerably across primary studies (Table 4). Authors of reviews reported Pilates exercise sessions of 15–60 minutes duration, 1–7 times per week, for 10 days and up to 12 months [6-10]. There was also variation in the use of mat versus specialised equipment, and incorporation of home exercises [7]. Further research is therefore required to define the essential elements of Pilates exercise in people with chronic low back pain [10].

In terms of comparison treatments, usual care was defined differently across the primary studies [25,30,31]. This may have resulted in an inaccurate measurement of Pilates treatment effect as participants had variable types and amounts of “usual care” in both treatment and comparison groups [40]. Pereira et al. (2012) [8] and Lim et al. (2011) [7] also described physiotherapy interventions provided by O’Brien et al. (2006) [32] differently. Pereira et al. (2012) [8] considered physiotherapy to consist of only lumbar stabilisation exercise, however, Lim et al. (2011) [7] reported physiotherapy treatment as also involving other modalities. This may have also contributed to inaccurate measurements of treatment effect with the pooling of primary studies with variable comparison treatments [40].

Similar outcome measures were used in primary studies to assess the effect of Pilates on pain and disability. The majority of these outcome measures are validated for use in people with low back pain, and have been found to be reliable [33,34,41]. The different treatment effects reported by Lim et al. (2011) [7] and Pereira et al. (2012) [8], however, could relate to the use of different outcome measures for pain intensity provided for Anderson (2005) [24].

Different findings between meta-analyses could also relate to different grouping of primary studies. For example, Aladro-Gonzalvo et al. (2012) [10] considered alternative exercise to Pilates to be a minimal intervention, while Lim et al. (2011) [7] and Pereira et al. (2012) [8] did not. Classifying alternative exercise to Pilates as a “minimal intervention” could be considered inappropriate as exercise has been found to reduce pain and disability in people with CLBP [38]. Effect sizes for Pilates may therefore be more conservative in Aladro-Gonzalvo et al. (2012) [40].

### Included primary studies

A comparison of included primary studies in reviews was undertaken as incorporating additional evidence can lead to different results [42]. Nine of the primary studies were available at the time of publication of the first systematic review [6]. La Touche et al. (2008) [6] and Posadzki et al. (2011) [9], however, chose to exclude unpublished primary studies and abstract articles (Table 7). This means that the findings of these reviews could be inflated as unpublished studies often have outcomes that are less positive or statistically insignificant [43].

**Table 7:** Findings of systematic reviews: effectiveness of Pilates in people with chronic low back pain

**Systematic Review**	**Comparison to Pilates**	**Pain Levels**	**Disability**
1. Aladro-Gonzalvo et al. [10]	a) Minimal intervention e.g. no treatment, usual care, exercise	a) Reduction is statistically significant (SMD=−0.44, 95% CI −0.09,–0.80)	a) Reduction is not statistically significant (SMD = −0.28, 95% CI 0.07, –0.62)
	b) Other physiotherapeutic treatment e.g. massage, physiotherapy	b) Reduction is not statistically significant (SMD = 0.14, 95% CI 0.27, –0.56)	b) Reduction is statistically significant (SMD = −0.55, 95% CI −0.08,–1.03)
2. La Touche et al. [6]	* Usual care, back school exercise^+^	* Reduced	* Reduced
3. Lim et al. [7]	a) Minimal intervention e.g.no treatment, usual care, massage, physiotherapy	a) Reduction is statistically significant (SMD = −2.72, 95% CI −5.33, –0.11)	a) Reduction is not statistically significant (SMD =−0.74, 95% CI −1.81, 0.33)
	b) Other forms of exercise e.g. back school exercise, lumbar stabilisation	b) Reduction is not statistically significant (SMD =0.03, 95% CI −0.52, 0.58)	b) Reduction is not statistically significant (SMD = −0.41, 95% CI =−0.96, 0.14)
4. Pereira et al. [8]	a) Variable treatment e.g. no treatment, usual care, massage, physiotherapy	a) Reduction is not statistically significant (SMD =−1.99, 95% CI –4.35, 0.37)	a) Reduction is not statistically significant (SMD =−1.34, 95% CI –2.80, 0.11)
	b) Lumbar stabilisation exercise	b) Reduction is not statistically significant (SMD =−0.11, 95% CI −0.74, 0.52)	b) Reduction is not statistically significant (SMD =−0.31, 95% CI −1.02, 0.40)
5. Posadzki et al. [9]	* Usual care, back school exercise	* Unknown, evidence is inconclusive	* Unknown, evidence is inconclusive

Note : SMD - standardised mean difference; 95% CI - 95% confidence level.

^*+*^ Back school exercise includes respiratory and postural education, muscle strengthening and mobilisation exercise [7,23].

In contrast, Aladro-Gonzalvo et al. (2012) [10], Lim et al. (2011) [7] and Pereira et al. (2012) [8] included several unpublished theses and an abstract study in their reviews (Table 5). These reviews, then, are likely to have less publication bias and more realistic findings [43]. Pereira et al. (2012) [8] also excluded primary studies that had a high risk of bias as defined by the Cochrane Back Review Group [36]. This review’s findings may therefore have greater credibility than other reviews [44].

The meta-regression analysis undertaken by Aladro-Gonzalvo et al. (2012) [10] did not identify any predictor variables that could explain differences in treatment effects across studies. This is not surprising, however, as the power of meta-regression was limited due to too few studies, and their heterogeneity [23,45]. The rationale for examining several co-variants is also questionable, and aggregation bias likely as client-specific characteristics such as the duration of complaint were taken from the mean results of studies rather than individual statistics [23,46,47].

### Level of evidence

The NHMRC level of evidence of all reviews was lower than expected for systematic reviews due to the inclusion of primary studies that were not randomised controlled trials. Aladro-Gonzalvo et al. (2012) [10], La Touche et al. (2011) [6], Lim et al. (2011) [7], and Posadzki et al. (2011) [9] represent the lowest level of evidence (Level IV) on the NHMRC hierarchy [1]. This is because these reviews included Donzelli et al. (2006) [27], a parallel case series article. Pereira et al. (2012) [8], however, represents Level III evidence on the NHMRC hierarchy as this review included only pseudo-randomised and randomised controlled trials. This means the findings of all reviews may contain bias related to the methodological design of primary studies, but Pereira et al. (2012) [8] may be less biased than other reviews [1,48].

### Methodological quality

The methodological quality of reviews was analysed to assist in the interpretation of findings [5]. The R-AMSTAR provided a numerical score of methodological quality for each review based on AMSTAR criteria [11]. The AMSTAR is reported as valid and reliable in assessing methodological quality of systematic reviews [5,15,21,22]. The inter-rater agreement for R-AMSTAR scores remained “substantial” as indicated by a kappa score of 0.78, 95% confidence interval: 0.71-0.85 [37]. This is similar to other scores reported for AMSTAR in the literature [22].

R-AMSTAR scores provide an indication of level of bias in review findings with high scores indicating greater credibility of findings [15]. Findings of Aladro-Gonzalvo et al. (2012) [10] which scored 37/44, can therefore be considered to be the most robust in relation to the methodological quality of systematic reviews. Examining individual item scores with the R-AMSTAR, however, is also critical to identify factors that influence the credibility of findings.

La Touche et al. (2008) [6] and Pereira et al. (2012) [8], for example, did not consider the methodological quality of primary studies in forming their conclusions. This is despite significant methodological flaws being identified in primary studies, such as small sample sizes, baseline differences between treatment and control groups, high drop-out rates, lack of assessor blinding, and intention to treat analyses [6,7,9]. The conclusions of La Touche et al. (2008) [6] and Pereira et al. (2012) [8], therefore, need to be interpreted with caution as these factors were not considered [49].

There is also a concern that the high R-AMSTAR scores of Aladro-Gonzalvo et al. (2012) [10], Lim et al. (2011) [7] and Pereira et al. (2012) [8] do not reflect the inappropriateness of conducting a meta-analysis. Aladro-Gonzalvo et al. (2012) [10], Lim et al. (2011) [7] and Pereira et al. (2012) [8] pooled the results of primary studies that had similar comparison groups, but different treatment protocols, outcome measures, and timing of re-assessments (Table 2). This clinical heterogeneity should have indicated that conducting a meta-analysis was inappropriate [36]. This is because pooling heterogeneous studies can produce inaccurate treatment effects [15,50,51].

Significant statistical heterogeneity (for example I^2^>60%) was also reported in both reviews when Pilates was compared to usual care [7,8,10]. This again suggests meta-analysis is inappropriate [52]. Using a random effects model to compensate for heterogeneity may have assisted to improve the accuracy of findings, but it does not explain or remove the primary study differences [36]. Moreover, combining two few primary studies in a meta-analysis can also produce misleading results [53]. The findings of Aladro-Gonzalvo et al. (2012) [10], Lim et al. (2011) [7] and Pereira et al. (2012) [8] therefore need to be interpreted carefully due to the small number and heterogeneity of primary studies.

## Conclusion

We are in agreement with Posadzki et al. (2011) [9], that there is inconclusive evidence that Pilates is effective in reducing pain and disability in people with CLBP. This conclusion relates to the insufficient number and methodological quality of available primary studies, rather than the methodological quality of reviews. These findings contrast to other review conclusions where Aladro-Gonzalvo et al. (2012) [10], La Touche et al. (2008) [6] and Lim et al. (2011) [7] report some effectiveness of Pilates, and Pereira et al. (2012) report no effectiveness.

Subsequent systematic reviews need to ensure that conclusions consider the methodological design and quality of primary studies. Meta-analyses and meta-regression analyses should also not be conducted when there is significant clinical and statistical heterogeneity across studies, and when primary studies are few in number. The Revised Assessment of Multiple Systematic Reviews provides a useful method of appraising the methodological quality of systematic reviews. Individual item scores, however, need to examined, in addition to total scores. This will ensure that significant methodological flaws are not missed, and results of reviews are interpreted appropriately.

## Abbreviations

AMSTAR: Assessment of Multiple Systematic Reviews; CINAHL: Cumulative index to Nursing and Allied Health Literature; CLBP: Chronic Low Back Pain; ICF: International Classification of Health Functioning, and Disability; NHMRC: National Health and Medical Research Council; PEDRO: Physiotherapy Evidence Database; PRISMA: Preferred Reporting Items for Systematic Review and Meta-Analyses; R-AMSTAR: Revised Assessment of Multiple Systematic Reviews; SF-36: 36 Item Short Form Health Survey.

## Competing interests

There are no financial or non-financial competing interests for any of the authors of this review.

## Authors’ contributions

CW designed, conducted, and drafted this systematic review with advice and guidance from AB, GSK, and PM. CW and BM evaluated the level of evidence and methodological quality of systematic reviews. AB acted as the third reviewer to obtain a consensus regarding methodological scores of reviews where there was disagreement. All authors reviewed the content of the manuscript prior to submission for its intellectual content. All authors read and approved the final manuscript.

## Authors’ information

CW is a registered physiotherapist undertaking doctoral studies at the University of Western Sydney under the supervision of AB, GSK, and PM. AB is an Associate Professor of Physiotherapy at Griffith University, however, the majority of this work was undertaken while AB was the Foundation Associate Professor and Head of Physiotherapy program at the University of Western Sydney. GSK is the Professor of Health Science and Dean of Science and Health, PM a Senior Lecturer, and CW a Lecturer in the School of Science and Health at the University of Western Sydney. BM is a registered physiotherapist and doctoral student at the University of Western Sydney.

## Pre-publication history

The pre-publication history for this paper can be accessed here:

http://www.biomedcentral.com/1471-2288/13/7/prepub
